# Translatability of findings from cynomolgus monkey to human suggests a mechanistic role for IL-21 in promoting immunogenicity to an anti-PD-1/IL-21 mutein fusion protein

**DOI:** 10.3389/fimmu.2024.1345473

**Published:** 2024-01-26

**Authors:** Mark A. Kroenke, Marta Starcevic Manning, Christina L. Zuch de Zafra, Xinwen Zhang, Kevin D. Cook, Michael Archer, Martijn P. Lolkema, Jin Wang, Sarah Hoofring, Gurleen Saini, Famke Aeffner, Elizabeth Ahern, Elena Garralda Cabanas, Ramaswamy Govindan, Mun Hui, Shalini Gupta, Daniel T. Mytych

**Affiliations:** ^1^ Clinical Immunology, Amgen, Thousand Oaks, CA, United States; ^2^ Translational Safety & Bioanalytical Sciences, Amgen, Thousand Oaks, CA, United States; ^3^ Translational Safety & Bioanalytical Sciences, Amgen, South San Francisco, CA, United States; ^4^ Clinical Pharmacology, Modeling, and Simulation, Amgen, South San Francisco, CA, United States; ^5^ Pharmacokinetics and Drug Metabolism, Amgen, South San Francisco, CA, United States; ^6^ Global Safety, Amgen, Thousand Oaks, CA, United States; ^7^ Early Development, Amgen, Thousand Oaks, CA, United States; ^8^ Medical Oncology, Monash Health, Clayton, VIC, Australia; ^9^ Research Unit, Hospital Universitario Vall d’Hebron, Barcelona, Spain; ^10^ Division of Hematology and Oncology, Washington University Medical School, St. Louis, MO, United States; ^11^ Chris O’Brien Lifehouse, Camperdown, NSW, Australia

**Keywords:** PD-1, IL-21, immunogenicity, anti-drug antibodies, mutein, IgE

## Abstract

AMG 256 is a bi-specific, heteroimmunoglobulin molecule with an anti-PD-1 antibody domain and a single IL-21 mutein domain on the C-terminus. Nonclinical studies in cynomolgus monkeys revealed that AMG 256 administration led to the development of immunogenicity-mediated responses and indicated that the IL-21 mutein domain of AMG 256 could enhance the anti-drug antibody response directed toward the monoclonal antibody domain. Anti-AMG 256 IgE were also observed in cynomolgus monkeys. A first-in-human (FIH) study in patients with advanced solid tumors was designed with these risks in mind. AMG 256 elicited ADA in 28 of 33 subjects (84.8%). However, ADA responses were only robust and exposure-impacting at the 2 lowest doses. At mid to high doses, ADA responses remained low magnitude and all subjects maintained exposure, despite most subjects developing ADA. Limited drug-specific IgE were also observed during the FIH study. ADA responses were not associated with any type of adverse event. The AMG 256 program represents a unique case where nonclinical studies informed on the risk of immunogenicity in humans, due to the IL-21-driven nature of the response.

## 1 Introduction

Inhibition of the PD-1/PD-L1 T cell checkpoint pathway has been established as an effective and generally well-tolerated approach to stimulating an immune response to tumor cells (1). While improved objective responses and/or improved overall survival have been observed in numerous patients, a significant subset of patients do not benefit from monotherapy (2). Consequently, various types of combination approaches are being investigated, including recombinant human IL-21 (rhIL-21).

IL-21 is a pleiotropic cytokine with the potential to catalyze a variety of downstream signaling events (3). In the context of immunotherapy for oncology indications, it has the potential to synergize with blockade of PD-1/PD-L1 by supporting a gene expression profile consistent with immature effector CD8 T cells (4). Furthermore, the combination of PD-1 blockade with IL-21 has shown remarkable efficacy in mouse tumor models, largely by enabling enhanced infiltration of CD8 T cells into the tumor (5).

To capitalize on the synergistic therapeutic potential of PD-1/PD-L1 inhibition and IL-21 signaling, a bifunctional fusion protein was created. AMG 256 is a fully human, aglycosylated heteroimmunoglobulin molecule, with 2 different heavy chains held together by charge pair mutations. One heavy chain is linked to an affinity-attenuated, monovalent, human IL-21 mutein. The monoclonal antibody domain (clone 22D4) is specific for PD-1. AMG 256 was designed to deliver an IL-21 signal specifically to PD-1^+^ CD8 T cells, while simultaneously inhibiting PD-1 signaling (6).

The nonclinical safety and pharmacokinetic (PK) profile of AMG 256 was evaluated in exploratory and Good Laboratory Practice (GLP) PK/pharmacodynamic (PD) and toxicology studies in cynomolgus monkeys because it binds with similar high affinity to the extracellular domains of human and cynomolgus monkey PD-1, but not to rodent PD-1. This is consistent with expectations of species specificity based on the protein sequence similarity which is 96% for cynomolgus monkey PD-1, but only 62.4% for mouse PD-1, relative to human PD-1 (7). Additionally, AMG 256 blocks the interaction of the human and cynomolgus monkey receptors with the human ligands, PD-L1 and PD-L2 (data not shown). Furthermore, the amino acid sequence homology between human and cynomolgus monkey IL-21 receptor (IL-21R) is 96.5% (7), but between human and mouse is only 62% (8).

A phase 1, first-in-human (FIH) study was designed to assess the safety, tolerability, pharmacokinetic, and pharmacodynamic properties of AMG 256 in patients with advanced solid tumors. Nonclinical studies had indicated that fusion of the IL-21 mutein domain to the monoclonal antibody domain could result in enhanced anti-drug antibody (ADA) responses, and potential class switching to the IgE isotype. Consequently, the FIH study was specifically designed to mitigate the risk of immunogenicity and hypersensitivity.

## 2 Materials and methods

### 2.1 Nonclinical study designs

A series of Investigational New Drug (IND)-enabling PK/PD and toxicology studies were conducted in cynomolgus monkeys at AAALAC-accredited facilities. All procedures conducted in animals complied with the Animal Welfare Act, the Guide for the Care and Use of Laboratory Animals, and the Office of Laboratory Animal Welfare. Protocols were approved by the applicable Institutional Animal Care and Use Committees.

In an exploratory PK/PD study, AMG 256 (5 mg/kg) or 22D4 (5 mg/kg) were administered to male cynomolgus monkeys by IV bolus injection on days 1 and 15 (n=4/group). A third group was dosed with 22D4 (5 mg/kg) on days 1 and 15 and rhIL-21 (0.1 mg/kg) on days 1, 4, 7, 15, 18, and 21. Blood samples for evaluation of serum chemistry parameters were obtained predose on day 1 and at 24 and 168 hours postdose, and predose on day 15 and at 24 and 168 hours postdose. Serum samples for the evaluation of PK were obtained on day 1 at 5 and 15 minutes and 1, 24, 72, 120, 168, and 240 hours post dose, and predose on day 15 and at 5 and 15 minutes and 1, 24, 72, 120, 168, and 240 hours post dose. Serum samples for evaluation of immunogenicity were obtained predose on day 15 (336 hours) and on day 25 (576 hours) after the first dose administration on day 1.

In an exploratory toxicology study, male cynomolgus monkeys were administered 3 weekly doses of AMG 256 by IV bolus injection at 10 or 30 mg/kg (n=3/group). Blood samples for the evaluation of clinical chemistry and hematology were collected prestudy and on days 2, 8, 9, 15, and 19. Blood samples for evaluation of coagulation parameters were collected prestudy and on days 2, 9, and 19. Serum samples for the evaluation of toxicokinetics (TK) were collected at 5 minutes and 1, 24, 72, 96, and 168 hours after the day 1 dose administration; at 5 minutes and 96 and 168 hours after the day 8 dose administration; and at 5 minutes and 1, 24, 72, and 96 hours after the day 15 dose administration. Serum samples for the evaluation of immunogenicity were obtained predose on days 1 and 8, and on day 19. Plasma samples for the analysis of the complement split products Bb, C3a, C5a, and sC5b9 and serum samples for CH50 analysis were collected prestudy and 30 minutes postdose on days 1, 8, and 15. Necropsy was conducted on day 19.

In a GLP toxicology study, male and female cynomolgus monkeys were administered 4 weekly doses of AMG 256 by IV bolus injection at doses of 0, 6, 30, or 150 mg/kg (n=3/sex/group). Blood samples for the evaluation of clinical chemistry and hematology were obtained prestudy and on days 2, 9, 16, 23, and 29. Blood samples for evaluation of coagulation parameters were collected prestudy and on day 29. Serum samples for evaluation of toxicokinetics were obtained predose on days 1, 15, and 22; 15 minutes postdose on days 1, 8, 15, and 22; and 4, 24, 48, 72, 96, and 168 hours postdose on days 1 and 22. Samples for evaluation of immunogenicity were obtained at baseline, predose on days 8, 15, and 22, and on day 29. Necropsy was conducted on day 29.

### 2.2 Nonclinical assays

Quantitation of AMG 256 and 22D4 in cynomolgus monkey serum was performed using electrochemiluminescent (ECL)-based immunoassays. For the exploratory PK/PD and toxicology studies, the method used biotinylated PD-1 (R&D Systems; Minneapolis, MN) as the capture reagent and ruthenylated mouse anti-human Fc (Amgen Inc.; Thousand Oaks, CA) as the detection reagent. For the GLP toxicology study, the validated method used rhPD-1 (Amgen Inc.) as the capture regent and ruthenylated mouse anti-human Fc (Amgen Inc.) as the detection reagent. Analyte serum concentrations were interpolated from standard curves using the corresponding analytes.

Anti-AMG 256 IgG was assessed in the nonclinical studies using the universal indirect species-specific assay (UNISA) (9). AMG 256 or 22D4 (if applicable) was coated on a bare Mesoscale Discovery plate (MSD; Rockville, MD), then washed and blocked. Serum samples were diluted and incubated on the drug-coated plate before washing and addition of a ruthenylated anti-cyno IgG detection reagent. Plates were washed and ECL signal was read using an MSD plate reader. Specificity was confirmed by incubating diluted serum samples with excess drug.

Anti-AMG 256 IgE was assessed in the nonclinical studies using an ECL-based immunoassay. Anti-cynomolgus monkey IgE antibody was coated on a standard bare MSD plate, then washed and blocked. Diluted serum samples were added to the plate to capture total IgE antibodies. Plates were washed and ruthenylated AMG 256 was utilized to detect drug-specific IgE bound to the plate. Specificity was confirmed by adding excess unlabeled AMG 256 to the detection reagent.

Several assays were performed to evaluate complement activation following 3 weekly doses of AMG 256 in the exploratory toxicology study. CH50 was measured by a hemolytic assay based on lysis of antibody-coated sheep red blood cells due to activation of complement on the cell’s surface. Serial dilutions of the test specimen were mixed with equal volumes of sheep red blood cells and the amount of hemoglobin released when the target cells were lysed by the action of complement was measured. Serial dilutions of a human serum standard with known CH50 activity were used to establish its 50% lysis point; each specimen was diluted in the same manner, and individual 50% lysis points were determined by linear regression.

Bb, C3a, and soluble C5b-9 (sC5b-9) were measured by ELISA. Assay standards, controls, and test specimens were diluted and placed in duplicate into wells precoated with a monoclonal antibody against Bb or C3a. After washing to remove unbound proteins, a second anti-Bb, anti-C3a, or sC5b-9 antibody conjugated to horseradish peroxidase was added; after an appropriate incubation time and washing, a chromogenic substrate was added, and the wells were assessed spectrophotometrically.

C5a was assessed using a competitive radioimmunoassay. Cross-reacting high molecular weight antigen (native C5) was removed from specimens by precipitation, and radiolabeled (^125^I) C5a antigen of known concentration was mixed with the plasma specimen. The mixtures were precipitated by adding a limited quantity of polyclonal human C5/C5a antibody. As C5a from the specimen competed with labeled C5a for binding to the antibody, the amount of radiolabeled antigen that precipitated was inversely proportional to the amount of C5a antigen present in the specimen.

### 2.3 Human *in vitro* T cell assay

Donors were recruited at phase 1 clinical trial units and selected to represent the global frequency of HLA-DRB1 alleles. A dendritic cell and T cell co-culture (DC:T) assay was performed by Lonza (Saffron Walden, UK). Briefly, monocytes were isolated from PBMCs through positive selection, and differentiated into immature dendritic cells using GM-CSF and IL-4. Immature dendritic cells were loaded with test proteins and matured using TNFα and IL-1β. Autologous CD4 T cells were isolated from PBMCs using negative selection and co-cultured with the mature dendritic cells for 6 days before CD3^+^ CD4^+^ Edu^+^ cells were measured by flow cytometry, with each condition carried out in 6 replicates. The stimulation index (SI) was calculated by dividing the test condition by the media alone control (baseline). A donor is generally considered a responder if SI ≥ 2.

### 2.4 FIH study design

The phase 1 study (NCT04362748) was designed to evaluate the safety, tolerability, pharmacokinetics, and pharmacodynamics of AMG 256 in patients with advanced solid tumors. The study was a non-randomized, open-label study with AMG 256 administered by intravenous (IV) infusion on days 1, 8, 15, and 22 of every 28-day cycle (QW dosing), or days 1 and 15 of every 28-day cycle (Q2W dosing). Dose escalation began at 0.6 mg IV QW and increased up to 1400 mg IV QW, with two additional cohorts dosed with 1000 mg or 2000 mg AMG 256 Q2W. Immunogenicity was monitored every week for the first cycle, every 2 weeks for cycle 2, and at the start of each cycle for cycles 3 and beyond. Subjects were observed for 24 hours after each infusion during cycles 1 and 2, and for 1 hour after each infusion during cycle 3 and all subsequent cycles. Dosing was staggered for the first 2 cycles to minimize the potential for multiple subjects experiencing hypersensitivity reactions on the same day. Informed consent was obtained from all subjects before participation.

### 2.5 Anti-drug antibody methods

Two different antibody assay methods were validated; one to detect all anti-AMG 256 antibodies in human serum and another to detect only antibodies that bind to endogenous human IL-21. The cut points for both assays were calculated from 30 healthy donor serum samples and 30 donor serum samples from patients with solid tumors, in accordance with regulatory guidance. Both assays were composed of screening and confirmatory components. Samples with a signal to noise (S/N) ratio higher than the assay cut point in the screening assay were analyzed with excess AMG 256 or IL-21 in the confirmatory assay to assess specificity. Percent depletion was calculated by subtracting the mean electrochemiluminescent (ECL) value of the treated specimen from the mean ECL value of the untreated specimen and dividing by the untreated specimen mean ECL value.

Anti-AMG 256 antibodies were measured using a validated, affinity capture elution (ACE) method. Maxisorp plates were coated with AMG 256, washed, and blocked. Samples were diluted 1:10 in 300 mM acetic acid to enable antibody-drug complex dissociation prior to analysis. The coated and blocked plates were washed, 1 M Tris pH 9.5 was added to each well, followed immediately by acid-diluted serum samples. Plates were incubated overnight to allow the coated drug to capture ADA from the neutralized sample. Plates were then washed, and 300 mM acetic acid was added to the plate to elute the bound ADA. The acid eluted samples were then neutralized with 1 M Tris pH 9.5, added to bare MSD high bind plates and allowed to incubate. The plates were then washed and blocked. Next, untreated or drug-treated detection buffer containing ruthenylated AMG 256 and excess unlabeled drug (confirmatory assay only) was added to the plates. Lastly, the plates were washed and tripropylamine MSD read buffer was added to each well. An electrical current was placed across the plate-associated electrodes using an MSD plate reader, resulting in a series of electrically induced oxidation-reduction reactions involving ruthenium and tripropylamine. The overall assay sensitivity was 3.2 ng/mL of anti-AMG 256 polyclonal antibody. At 100 ng/mL of anti-AMG 256 antibody, the assay could tolerate at least 200 µg/mL of excess AMG 256.

Antibodies against endogenous IL-21 were measured using a validated, ECL bridging method. Prior to analysis, samples were treated with 300 mM acetic acid (1:40) to enable antibody-IL-21 complex dissociation. Then, acid treated samples were neutralized and incubated overnight in a mixture of biotinylated-IL-21 and ruthenylated-IL-21 (and excess unlabeled IL-21 in the confirmatory assay). ADA present in serum samples form a bridge between the two IL-21 conjugates. The formed antibody complex was captured on a blocked streptavidin plate, washed, and analyzed on a plate reader where signal was produced from an electrically induced oxidation-reduction reaction involving ruthenium and tripropylamine. The overall assay sensitivity was 4.3 ng/mL of anti-IL-21 monoclonal antibody. At 100 ng/mL of anti-IL-21 antibody, the assay could tolerate at least 250 µg/mL of excess AMG 256.

Anti-AMG 256 IgE antibodies were detected using an ECL-based method consisting of a screening and confirmatory assay. Total IgE antibodies from serum samples (diluted 1:40 in assay diluent) were captured onto a bare standard-bind MSD plate coated with anti-human IgE capture antibody. The MSD plate was washed and a detection reagent containing either ruthenylated AMG 256 (screening assay) or ruthenylated AMG 256 with excess unlabeled AMG 256 (confirmatory assay) was added to the plate. The plate was washed and tripropylamine-containing MSD read buffer was added to each well. Using an MSD plate reader, an electrical current was placed across the plate-associated electrodes, inducing a series of reactions involving ruthenium and tripropylamine and resulting in an ECL signal. Sample results were expressed as a S/N ratio, calculated by dividing sample signal by the signal of the negative control. Samples with a S/N greater than or equal to the screening assay cut point that demonstrated signal inhibition greater than or equal to the confirmatory cut point in the presence of excess unlabeled AMG 256 were considered positive for AMG 256-specific IgE antibodies. Cut points were established according to regulatory guidance using data from 103 individual donor serum samples (61 healthy and 42 solid tumor), including 28 baseline samples from the FIH study. A chimeric mouse/human IgE antibody that binds a modified component of the AMG 256 Fc domain was used as a positive control to determine method parameters and monitor assay performance. Assay sensitivity was determined to be 111 pg/mL or 151 pg/mL in the screening or confirmatory assays, respectively. The screening assay was qualified to detect 0.5 ng/mL of anti-AMG 256 IgE antibody in the presence of 200 μg/mL of soluble AMG 256. The confirmatory assay was qualified to detect 0.5 ng/mL or 1 ng/mL of anti-AMG 256 IgE antibody in the presence of 50 μg/mL or 200 μg/mL, respectively, of soluble AMG 256. In addition, high concentrations of total IgE (up to 10 μg/mL) or AMG 256-specific IgG (up to 200 μg/mL) did not result in false positives or false negatives at 200 pg/mL of positive control.

### 2.6 Neutralizing antibody assay

A cell-based neutralizing antibody assay was developed and validated by PPD (Richmond, VA) to assess the ability of anti-IL-21 antibodies to neutralize endogenous IL-21. Briefly, Hut78 cells were stimulated with rhIL-21 and phosphorylation of STAT3 was measured using an MSD kit. In the presence of a neutralizing antibody, STAT3 phosphorylation was lost. The overall sensitivity of the assay was 128 ng/mL. At 0.5 µg/mL of excess AMG 256 in serum, the assay could detect at least 500 ng/mL of anti-IL-21 neutralizing antibodies.

### 2.7 Pharmacokinetic assay

AMG 256 was measured in human serum using a validated sandwich immunoassay. An anti-idiotype monoclonal antibody against the 22D4 domain was used for capture and a biotin conjugated anti-IL-21 monoclonal antibody was used for detection. The assay range was 10.0 to 1,000 ng/mL.

## 3 Results

### 3.1 *In vitro* T cell assay to assess sequence-based risk of immunogenicity

Prior to initiation of a clinical study, an *in vitro* T cell assay was performed to identify T cell epitopes and assess immunogenic risk of AMG 256. A DC:T assay format was utilized to eliminate the possibility that neutralization of PD-1 and/or IL-21 signaling could influence the result.

**Figure 1:**
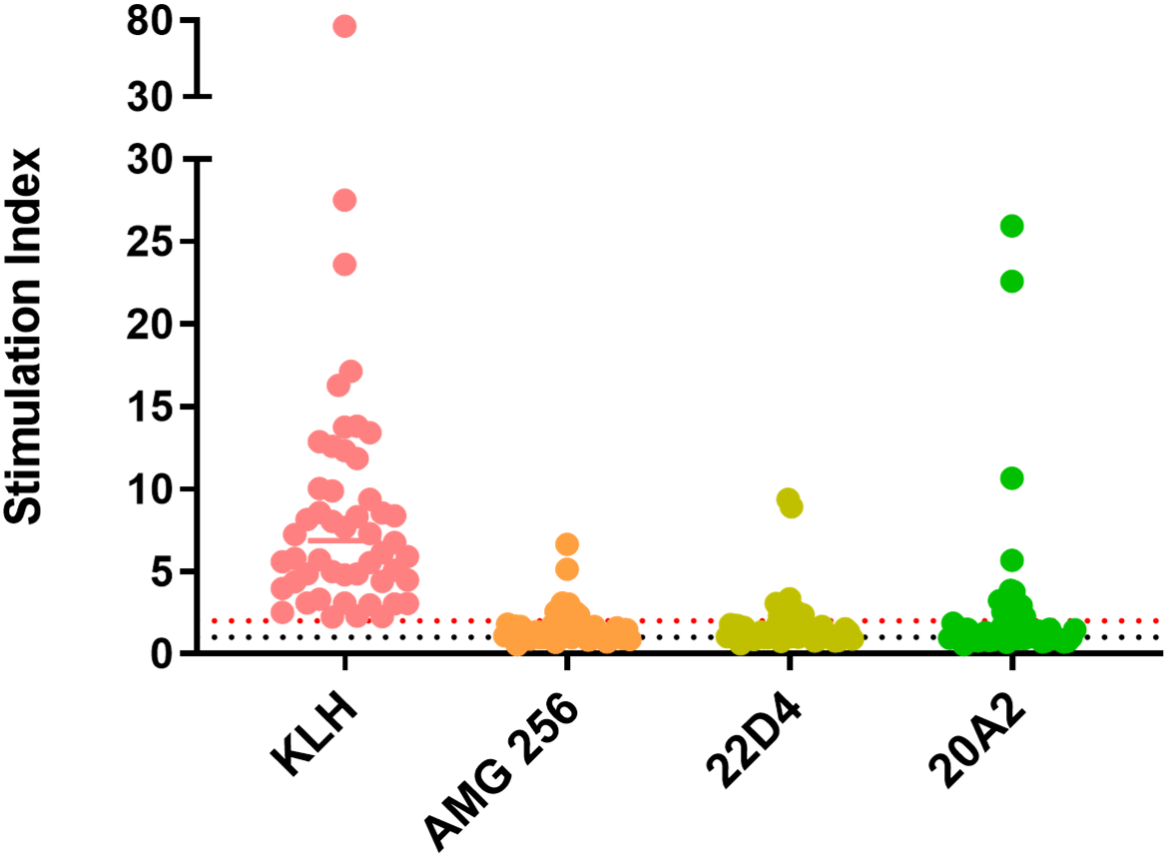
*In vitro* T cell assays did not suggest significant sequence-based risk of immunogenicity for AMG 256. A DC:T assay was performed with naïve donors representative of global HLA allele frequencies. Results are shown as stimulation index, or test protein divided by the baseline condition (media alone). Monocytes from 50 PBMC donors were differentiated into dendritic cells, loaded with test protein, and matured. Autologous CD4 T cells were isolated and co-cultured with mature dendritic cells presenting test protein agretopes for 6 days prior to assessment of CD4 T cell proliferation by flow cytometry. KLH was used as a positive control. Additional controls included 22D4 (AMG 256 MAb domain alone) and 20A2 (unrelated anti-PD-1 MAb). The black dashed line indicates an SI of 1 (no change from baseline) and the red dashed line indicates an SI of 2 (response).

All donors demonstrated an SI of greater than 2 in response to keyhole limpet hemocyanin (KLH) which was used as a positive control. The CD4 response to AMG 256 and the 22D4 monoclonal antibody domain alone was similar, with 10 of 50 and 11 of 50 donors, respectively, responding with an SI of greater than 2 (**Figure 1**). This indicates that the IL-21 mutein domain of AMG 256 contributes minimal sequence-based immunogenic risk. An additional anti-PD-1 clone, 20A2, was also tested as a potential alternative to 22D4. For 20A2, 14 of 50 donors had an SI greater than 2, with several large magnitude responses, indicating the risk of a T-dependent antibody response being elicited was lower for 22D4 than for 20A2. These data contributed to the selection of the 22D4 clone to comprise the antibody portion of AMG 256.

### 3.2 Clinical observations, loss of exposure, and ADA response in a cynomolgus monkey PK/PD study

The safety and PK profile of AMG 256 were characterized through a series of IND-enabling studies in cynomolgus monkeys, the most relevant nonclinical species for evaluation of the PK/PD and toxicity of AMG 256. In a PK/PD study, animals were dosed IV with AMG 256 or the 22D4 monoclonal antibody on days 1 and 15; a third group was dosed with 22D4 (5 mg/kg) on days 1 and 15 and rhIL-12 (0.1 mg/kg) on days 1, 4, 7, 15, 18, and 21. Within 6 to 10 minutes of administration of the second AMG 256 dose on day 15, 2 of 4 animals in the AMG 256 group showed signs of hypersensitivity-type reactions including decreased activity, dilated pupils, pale mucous membranes, salivation, and reddened facial skin; 1 of these 2 animals also transiently lost consciousness, had severe emesis, and was treated with diphenhydramine, dexamethasone, oxygen, oral honey, and intravenous fluids. Both animals were placed in incubators for observation. By approximately 6.5 hours after dosing, both animals appeared normal and were returned to their home cages without further need for medical treatment.

In the AMG 256 group, clinical chemistry alterations were generally small in magnitude, sporadic, and transient. They were consistent with hemolysis and/or altered hepatobiliary function included minimal to mild sporadic, transient increases in AST, ALT, ALP, and total bilirubin. Hematology was not evaluated in this study, therefore the relative contribution of hemolysis and/or hepatobiliary function to these changes cannot be determined. Inflammation was indicated by minimal to mild increases in CRP. Altered mineral and electrolyte metabolism was indicated by minimal sporadic, transient decreases in calcium, potassium, and phosphorus and minimal increases in sodium and chloride. None of the observations were considered adverse because they were of small magnitude, sporadic, and transient.

**Figure 2:**
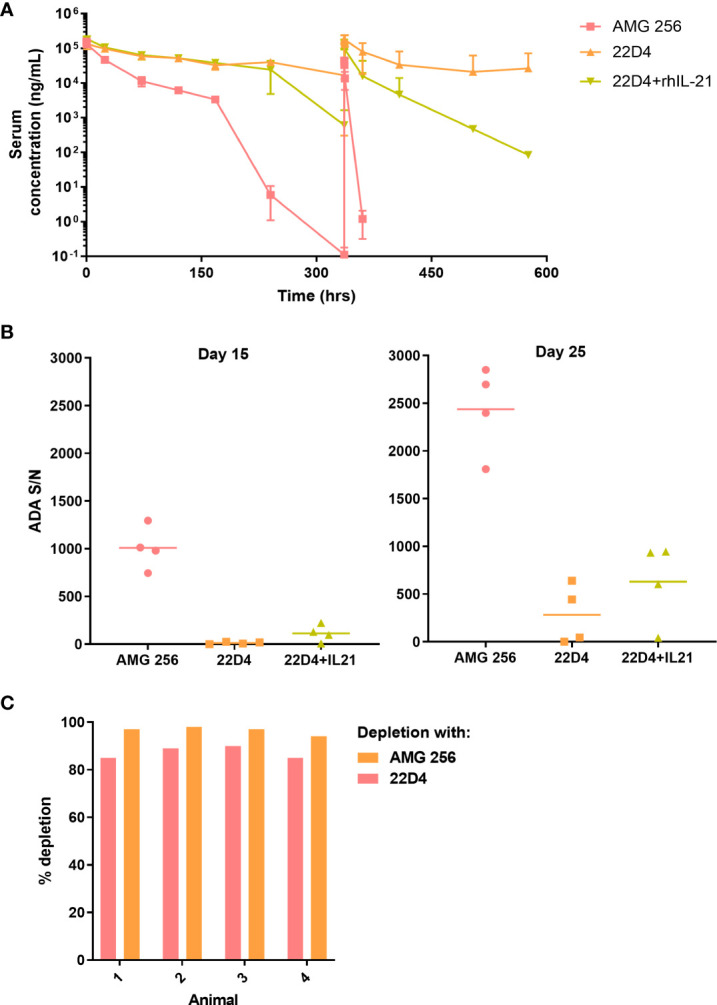
IL-21 mutein domain enhanced the antibody response to 22D4 in cynomolgus monkeys. Cynomolgus monkeys were dosed with 5 mg/kg AMG 256, 5 mg/kg 22D4, or 5 mg/kg 22D4 plus 0.1 mg/kg recombinant human IL-21. **(A)** AMG 256 or 22D4 serum levels were measured over time in each of the 3 treatment groups. **(B)** The ADA response in each dosing group was assessed on day 15 and day 25 by UNISA. **(C)** Domain characterization was performed on AMG 256 dosed animals at the day 25 time point. Serum samples were pre-treated with either AMG 256 or 22D4 and re-tested in the antibody assay. Percent depletion indicates the signal change from the pre-treated sample relative to the untreated sample.

While it was anticipated that 22D4 would have slightly higher exposure than AMG 256 (6), rapid loss of exposure was observed in the terminal phase of the first dose interval and upon subsequent dosing of AMG 256 (**Figure 2A**). All AMG 256-dosed animals were positive for anti-AMG 256 IgG on days 15 and 25 (hours 336 and 576 after the day 1 dose, respectively). Surprisingly, the antibody response in animals dosed with AMG 256 was uniformly and remarkably enhanced relative to animals dosed with 22D4 alone (**Figure 2B**). The antibody response magnitude for animals dosed with 22D4 and 22D4+rhIL-21 was similar, indicating that either the IL-21 mutations and/or the fusion to the 22D4 domain was required in order to observe this enhanced response.

One possible explanation for this observation was that the cynomolgus monkey response was primarily directed against the IL-21 domain of AMG 256, and consequently when this domain was not present, fewer antibodies were detected. In order to assess this, serum samples from animals dosed with AMG 256 were pre-treated with either AMG 256 or 22D4 and re-tested in the AMG 256 antibody assay. Assay signal was depleted to a similar extent with both AMG 256 and 22D4, demonstrating that the bulk of the antibody response was directed against the 22D4 domain of AMG 256 (**Figure 2C**). These data indicate that the IL-21 mutein domain of AMG 256 enhanced the 22D4 directed antibody response in cynomolgus monkey.

Based on the hypersensitivity-type clinical signs observed following the day 15 dose in 2 of 4 animals dosed with AMG 256, additional immunogenicity assessment was conducted to evaluate the presence of IgE isotype ADAs. It was important to assess this because there are plausible mechanisms by which the IL-21 mutein domain of AMG 256 could trigger class switching to IgE (10, 11). If this was observed in cynomolgus monkey studies, similar mechanisms may trigger drug-specific IgE in the FIH study, posing a safety risk to patients. All animals (4 of 4) administered AMG 256 were positive for IgE ADAs on days 15 and 25, except for a single animal that was IgE ADA-negative on day 15. IgE ADAs were not detected in any of the animals administered 22D4.

### 3.3 Activation of classical and alternative complement pathways in a cynomolgus monkey exploratory toxicology study

Following 3 weekly IV doses of AMG 256 to male cynomolgus monkeys at doses of 10 or 30 mg/kg, transient clinical signs including decreased activity, dilated pupils, pale skin, and loss of coordination were observed in 1 of 3 animals in the 30 mg/kg dose group. Anti-AMG 256 IgG antibodies were observed in all animals at the day 19 time point, resulting in loss of exposure similar to the PK/PD study (data not shown). A subset of animals was also tested for anti-AMG 256 IgE. This subset was composed of 1 animal from the 30 mg/kg group with potentially IgE-mediated clinical observations and 2 animals with no evidence of IgE (one from each group). The animal suspected of being IgE positive was confirmed positive for anti-AMG 256 IgE at day 19, along with one other animal from the 30 mg/kg group.

Based on the clinical observations and ADA responses in this study as well as the previous PK/PD study, samples were evaluated for evidence of complement pathway activation. Results were consistent with dose-dependent activation of both classical and the alternative complement pathways. CH50 values for all 10 mg/kg animals and 2 of 3 30 mg/kg animals remained relatively unchanged on days 1 and 8 when compared to prestudy levels. One of 3 animals in the 30 mg/kg group had a significant decrease in CH50 values, reflecting complement activation, following dosing on day 8, and all animals in both dose groups had dramatic decreases in CH50 values (down to 0 U/mL) following the third dose of AMG 256 on day 15. Increases in Bb, C3a, and sC5b-9 were observed in all animals, and the magnitude of the changes increased progressively over the course of the study. No notable changes were seen in C5a levels at all sampling timepoints; however, C5a has a relatively short half-life and is cleared rapidly from circulation, so it is possible that it was undetectable even in samples collected at 30 minutes post-dose (earliest sampling timepoint).

### 3.4 Consistent, robust anti-AMG 256 IgG response in a GLP cynomolgus monkey toxicology study

In a GLP toxicology study, cynomolgus monkeys were administered AMG 256 IV at 0, 6, 30, or 150 mg/kg once weekly for 4 weeks. Three animals displayed serious clinical signs, including pallor, weakness, petechia, hypothermia, and dehydration, leading to the unscheduled euthanasia of 2 animals in the 150 mg/kg dose group (on day 9 and day 11), and 1 animal in the 30 mg/kg dose group on day 22. The cause of moribundity in these animals was attributed to IgM ADA-mediated immune complex disease resulting in thrombocytopenia, consumptive coagulopathy, and circulatory collapse. Clinical pathology changes included decreased red blood cell (RBC) mass (hemoglobin, RBC count, and hematocrit), reticulocytes, and platelets; prolonged PT and aPTT and altered fibrinogen; decreased albumin; and/or increased C-reactive protein (CRP).

Clinical observations in several animals surviving to scheduled termination included emesis, discolored skin, salivation, weakness, hunched posture, decreased activity, diarrhea, and coughing/sneezing in individual animals at all dose levels. These signs were transient and generally occurred after at least 2 doses. AMG 256-related hematology alterations included decreased RBC mass with decreased then increased reticulocytes, decreased platelets, and decreased white blood cell count. AMG 256-related changes in clinical chemistry parameters included an acute phase response characterized by increased CRP, globulins, and triglycerides and/or decreased albumin and albumin/globulin ratio at all dose levels. Alanine aminotransferase, AST, and LDH were increased at ≥ 30 mg/kg and total bilirubin was increased at all dose levels. Alkaline phosphatase and GGT levels were decreased at 150 mg/kg and cholesterol was increased at all dose levels. Creatinine was increased at 150 mg/kg.

**Figure 3:**
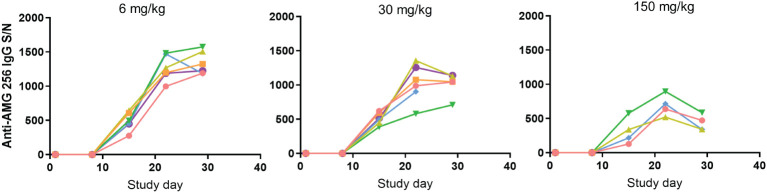
Antibody response in cynomolgus monkeys was robust and uniform. Cynomolgus monkeys were dosed with 6, 30, or 150 mg/kg AMG 256. UNISA was used to assess anti-AMG 256 IgG antibodies at baseline and 4 post-baseline time points. In the 30 mg/kg group, one animal was euthanized early on day 22, and in the 150 mg/kg group, two animals were euthanized early on days 9 and 11. Each color represents an individual animal.

AMG 256 exposure increased with dose, and an impact of anti-AMG 256 ADAs on exposure was observed in all animals except the 150 mg/kg dose group animal euthanized on day 11. At the day 8 predose time point, IgG ADAs were detected in 1 of 6 animals in the 6 mg/kg dose group; at the day 15 predose time point and onward, IgG ADAs were observed in all surviving animals. While it is not possible to differentiate immunogenicity driven by foreign human sequence from IL-21-driven immunogenicity, it was noted that every animal developed a robust anti-AMG 256 IgG response, and results were consistent across animals (**Figure 3**).

Some reduction in assay signal was observed as the dose of AMG 256 was increased, however, this is likely due to the impact of higher levels of circulating drug on S/N values, rather than a real reduction in antibody magnitude. This uniform ADA response was distinct from what is typically observed following administration of biotherapeutic molecules to cynomolgus monkeys, which is a varied ADA response driven by the diversity of cynomolgus monkey HLA alleles and T/B cell repertoires (9). These data further support the hypothesis that fusing an IL-21 mutein domain to a monoclonal antibody can enhance the ADA response to the antibody domain.

Based on observations of hypersensitivity and/or IgE ADA in prior cynomolgus monkey studies, together with the clinical observations in this study, drug-specific IgE was assessed. No animals in the control group tested positive for anti-AMG 256 IgE at any time point. IgE ADAs were detected in 2 animals at 6 mg/kg and 1 animal at 30 mg/kg on day 15, and by day 29 all 6 animals at 6 mg/kg, 4 of 6 animals at 30 mg/kg, and none of the animals at 150 mg/kg were IgE positive (**Table 1**). The two animals at 150 mg/kg that were euthanized at unscheduled time points were negative for both IgG and IgE ADAs at their last sampling time point on day 8 or day 9. The IgE response was low magnitude relative to IgG, as expected based on the relative concentration of IgE compared to IgG in serum. The decreased incidence of anti-AMG 256 IgE with increasing dose of AMG 256 was likely due to the concentration of AMG 256 in serum exceeding the drug tolerance of the IgE assay, and not an actual drop in ADA incidence.

**Table 1:** Drug-specific IgE results from GLP cynomolgus monkey study.

Group	Dose	Anti-drug IgE incidence	Positive sample peak S/N range
Group 1	Placebo	0/6	–
Group 2	6 mg/kg	6/6	1.97 – 3.61
Group 3	30 mg/kg	4/6	1.54 – 4.98
Group 4	150 mg/kg	0/6	–

### 3.5 Immunogenicity assessment in the AMG 256 FIH study

**Figure 4:**
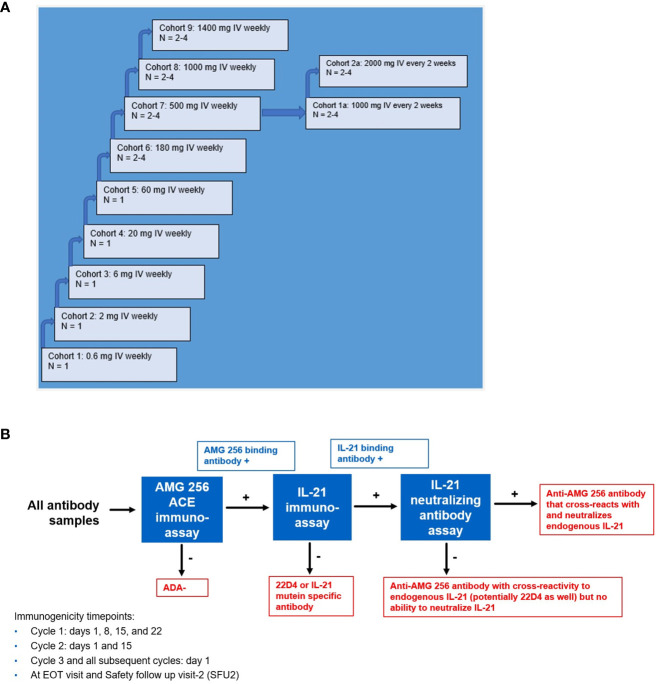
Study schema for first-in-human NCT04362748 and immunogenicity testing strategy. **(A)** NCT04362748 was designed as a multiple ascending dose phase 1 study to assess safety, tolerability, and PK/PD. **(B)** A comprehensive antibody testing strategy was implemented to monitor all anti-AMG 256 antibodies and the possibility of anti-AMG 256 antibodies that cross-react with and neutralize endogenous IL-21. End results are shown in red boxes. Results that require further characterization are shown in blue boxes.

Prior to initiating the FIH study, an immunogenicity risk assessment was conducted based on *in vitro* and nonclinical data as well as relevant literature. It was recognized that AMG 256 had the potential for mechanism of action (MOA)-driven immunogenicity as well as elicitation of drug-specific IgE. Due to these risks, a conservative approach to dosing human subjects was taken. Some examples of mitigations put in place included a low 0.6 mg starting dose (based on a minimally anticipated biological effect level [MABEL] approach), beginning the study with single subject dose escalation cohorts, staggering dosing in later multi-subject cohorts, long infusion times, and frequent ADA sampling (**Figure 4A**). Furthermore, the study was conducted at centers prepared with trained clinical personnel and resources to respond appropriately should severe hypersensitivity responses (i.e. anaphylaxis) occur. These safeguards ensured, among other things, that any AMG 256 hypersensitivity or anaphylactic reaction would be limited initially to one subject and mitigated to the extent possible.

Aside from the risk of IgG or IgE-mediated hypersensitivity, there was an additional risk that antibodies elicited to AMG 256 could cross-react with and neutralize endogenous IL-21. A tailored antibody monitoring strategy was devised to specifically address this risk. First, all antibody samples were screened for binding to AMG 256 using a sensitive and drug-tolerant ACE assay. If a sample tested positive for anti-AMG 256 antibodies, the sample was subsequently tested for antibodies that cross-react with endogenous human IL-21 using an independent assay. Lastly, if positive for binding to both AMG 256 and endogenous human IL-21, the sample was also tested for the ability to neutralize endogenous IL-21 in a cell-based assay (**Figure 4B**). A human anti-AMG 256 IgE assay was also developed in the event that hypersensitivity was observed and required further investigation.

**Figure 5:**
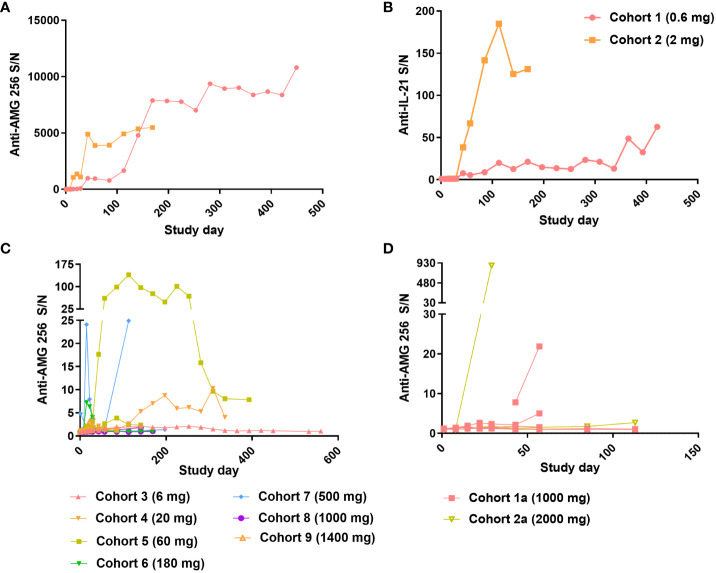
Robust antibody response observed at low doses of AMG 256. For cohorts 1 and 2, Anti-AMG 256 S/N **(A)** and anti-IL-21 S/N **(B)** are plotted by study day. For QW dosing cohorts 3 through 9 **(C)** and Q2W dosing cohorts 1a and 2a **(D)**, anti-AMG 256 S/N is shown by study day. Only subjects with at least one positive anti-AMG 256 antibody test result are shown, and individual subjects are shown for each cohort using the same color.

Upon dosing cohorts 1 and 2, a robust antibody response to AMG 256 was observed. For the cohort 1 subject, the magnitude of the response increased steadily throughout the first 11 cycles and likely saturated the immunoassay at a S/N of over 10,000 (**Figure 5A**). In cohort 2, the magnitude of the ADA response increased nearly 1000-fold between the 2nd and 3rd doses of AMG 256. The anti-AMG 256 antibodies observed in cohorts 1 and 2 also cross-reacted with endogenous IL-21 with much lower magnitude (**Figure 5B**), supporting the hypothesis that the IL-21 mutein was driving the antibody response to the 22D4 domain. Both subjects with anti-IL-21 antibodies also tested positive for neutralizing antibodies to endogenous IL-21 throughout most of their time on study (**Supplementary Table 1**).

As the study progressed to higher doses in cohorts 3 and beyond, the incidence of anti-AMG 256 antibodies remained high (**Table 2**); however, the magnitude of the antibody responses was significantly reduced compared to cohorts 1 and 2, and most subjects had S/N values in the single digits (**Figure 5C**). Only one subject in cohort 2a had an antibody response somewhat similar to that observed in cohorts 1 and 2 (**Figure 5D**).

**Table 2:** Incidence of treatment emergent^a^ binding and neutralizing antibodies in NCT04362748.

Cohort	Dose (IV)	Anti-AMG 256 antibody incidence	Anti-IL-21 antibody incidence	Neutralizing anti-IL-21 incidence
1	0.6 mg QW	1/1	1/1	1/1
2	2 mg QW	1/1	1/1	1/1
3	6 mg QW	1/1	0/1	–
4	20 mg QW	2/2	0/2	–
5	60 mg QW	3/3	0/3	–
6	180 mg QW	2/3	0/2	–
7	500 mg QW	3/4^b^	1/3	0/1
8	1000 mg QW	2/4	1/2	0/1
9	1400 mg QW	4/4	1/4	0/1
1a	1000 mg Q2W	5/6^c^	0/5	–
2a	2000 mg Q2W	4/4	1/4	0/1
	Total	28/33	6/28	2/6

^a^Treatment emergent defined as a subject who is antibody positive post-baseline with a negative or no result at baseline, or a subject who is positive at baseline with a >4-fold increase in antibody magnitude (S/N value) post-baseline.

^b^All subjects in cohort 7 were ADA positive, however, 1 subject was ADA positive at baseline and did not qualify as treatment emergent.

^c^One subject had a baseline antibody sample only (ADA negative) and was excluded from the table.

### 3.6 Impact of immunogenicity on exposure

**Figure 6:**
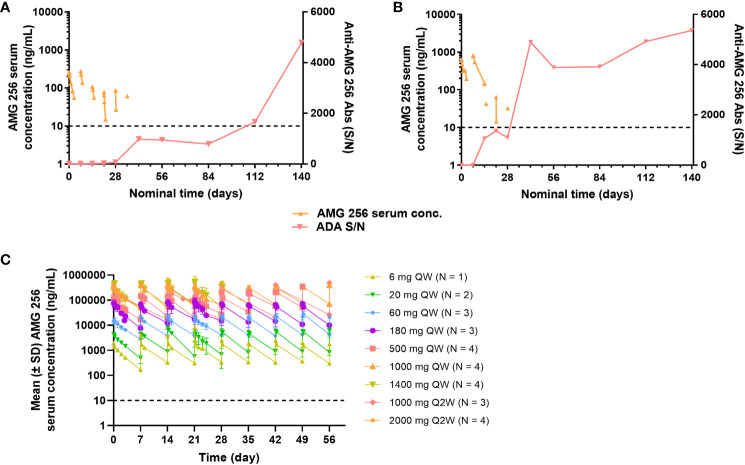
Anti-AMG 256 antibodies significantly impact exposure at low doses. The anti-AMG 256 antibody response S/N is plotted with serum concentration of AMG 256 for cohort 1 **(A)** and cohort 2 **(B)**. **(C)** Serum levels of AMG 256 are shown for cohort 3 and all subsequent cohorts. The lower limit of quantitation for the PK assay (10 ng/mL) is indicated by the dashed line.

Given the high incidence of anti-AMG 256 antibodies throughout the study, it was important to assess the impact on AMG 256 exposure. In cohorts 1 and 2, AMG 256 became undetectable in serum shortly after the development of anti-AMG 256 antibodies (**Figures 6A, B**). In all other cohorts, exposure to AMG 256 was maintained, with no apparent impact of anti-AMG 256 antibodies (**Figure 6C**). One subject in cohort 2a developed a larger magnitude anti-AMG 256 antibody response with no apparent impact on exposure, however, the subject left the study before exposure could be thoroughly evaluated. Only 4 subjects were antibody negative throughout the study, and all 4 had comparable AMG 256 exposures relative to the antibody positive subjects within the same cohort.

### 3.7 Anti-AMG 256 IgE detected at late time points

**Figure 7:**
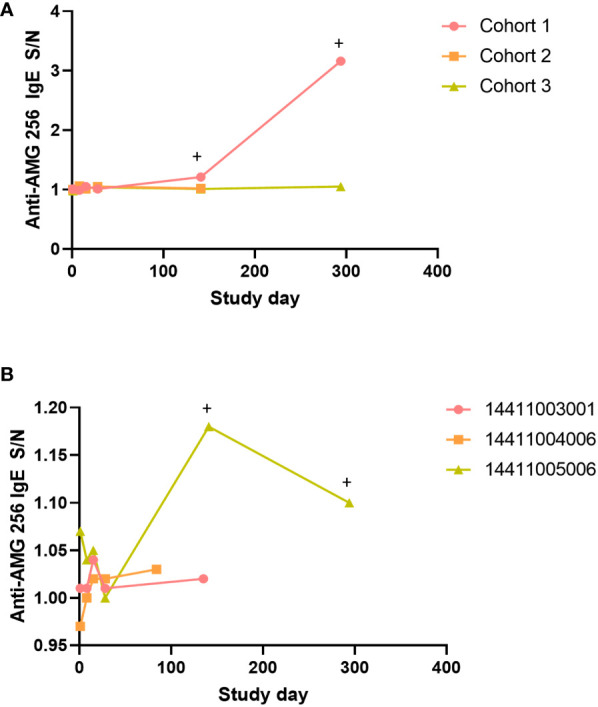
Anti-AMG 256 IgE antibodies detected in two subjects. The anti-AMG 256 IgE antibody response S/N is plotted for the single dose cohorts 1-3 **(A)** and cohort 5 **(B)**. Positive time points are denoted by a +. No anti-AMG 256 IgE positive subjects were identified in the other cohorts tested (4, 7, 1a).

A subset of subjects and time points in NCT04362748 were assessed for drug-specific IgE antibodies. This testing was performed to further characterize the antibody response, and was not triggered by an adverse event. Samples were selected to cover a range of doses, both early and late time points, and the full range of anti-AMG 256 antibody responses, based on the ACE assay. Post-baseline samples from a total of 16 subjects were analyzed. In the single subject cohorts 1-3, the cohort 1 subject was the only subject to test positive for anti-AMG 256 IgE, despite the cohort 2 subject exhibiting a similar, high magnitude anti-AMG 256 response by ACE (**Figure 7A**). Of the three subjects in cohort 5, only 1 tested positive for anti-AMG 256 IgE (**Figure 7B**). Both subjects positive for anti-AMG 256 IgE were positive at cycle 6 day 1 and cycle 12 day 1 (**Supplementary Table 2**). IgE responses were low magnitude, consistent with the low concentration of IgE in human serum relative to other immunoglobulin isotypes.

### 3.8 No impact of immunogenicity on safety observed

The impact of immunogenicity on safety was carefully assessed, especially in cohorts 1 and 2 given the presence of anti-IL-21 neutralizing antibodies. To determine what types of adverse events could potentially manifest as a result of an anti-IL-21 neutralizing antibody, a comprehensive literature search of compounds blocking IL-21 signaling was carried out. Several anti-IL-21 antibodies (12, 13) and an anti-IL-21R antibody (14) were identified and available clinical data were assessed. Overall, a decrease or loss of IL-21 signaling was well-tolerated, but theory and some studies suggested that loss of IL-21 signaling could cause immunosuppression relative to placebo. Based on this, adverse events in subjects with IL-21 neutralizing antibodies were carefully evaluated for any evidence of increased infections (**Table 3**). Subjects with neutralizing antibodies to IL-21 and subjects with anti-AMG 256 antibodies only (no cross-reactivity to endogenous IL-21) had adverse event profiles similar to antibody negative subjects, and there was no discernable impact of immunogenicity on safety.

**Table 3:** Treatment-related, treatment-emergent adverse events and immunogenicity in NCT04362748^a,b^.

Cohort	1^d,e^ (n=1)	2^d^ (n=1)	3 (n=1)	4 (n=2)	5^e^ (n=3)	6 (n=3)		7 (n=4)	8^f^ (n=4)		9 (n=4)	1a (n=7)		2a (n=4)
ADA status^c^	Pos	Pos	Pos	Pos	Pos	Pos	Neg	Pos	Pos	Neg	Pos	Pos	Neg	Pos
All treatment-related, treatment-emergent AEs	1	1	1	2	3	2	1	1	1	1	2	4	2	4
Blood and lymphatic system disorders	0	0	0	0	0	1	0	0	0	0	1	1	1	1
Endocrine disorders	0	0	0	2	2	1	0	0	0	1	0	1	0	1
Eye disorders	0	0	0	0	0	0	0	0	1	0	0	0	0	0
Gastrointestinal disorders	0	0	0	0	1	0	0	0	0	0	0	1	1	1
General disorders and administration site conditions	0	0	1	2	0	2	0	0	0	0	1	2	0	1
Hepatobiliary disorders	0	0	0	0	0	0	0	0	1	0	0	0	0	0
Immune system disorders	0	0	0	0	1	0	0	0	0	0	1	0	0	0
Infections and infestations	0	0	0	0	1	0	0	0	0	0	0	0	0	0
Injury, poisoning and procedural complications	0	0	0	0	0	0	0	0	0	1	0	0	0	0
Investigations^g^	0	1	1	1	1	1	1	0	1	0	0	1	0	1
Metabolism and nutrition disorders	0	0	0	0	0	1	0	0	0	0	0	0	0	0
Musculoskeletal and connective tissue disorders	0	0	0	0	1	0	0	0	1	0	0	0	1	3
Neoplasms benign, malignant, and unspecified	0	0	0	0	0	0	0	0	1	0	0	0	0	0
Nervous system disorders	1	0	0	1	0	0	0	0	0	0	0	0	0	1
Renal and urinary disorders	0	0	0	0	0	0	0	0	0	0	0	0	1	0
Skin and subcutaneous tissue disorders	0	0	0	1	3	0	0	1	1	1	0	0	0	0
Vascular disorders	0	0	0	0	1	0	0	0	0	0	0	0	0	0

^a^Subject incidence of adverse events by System Organ Class (SOC), for adverse events considered by the study investigator to be at least possibly related to investigative product.

^b^Medical Dictionary for Regulatory Activities version 25.1 (MedDRA).

^c^Defined as positive for anti-AMG 256 antibodies at any time.

^d^One subject in each of cohorts 1 and 2 developed neutralizing antibodies to endogenous IL-21. In theory, these two subjects may have been considered at increased risk of experiencing infection events. However, these two subjects experienced no adverse events considered causally related to investigative product within the SOC “Infections and Infestations.”.

^e^Each of cohorts 1 and 5 included one subject positive for anti-AMG 256 IgE. In theory, these two subjects may have been considered at increased risk of experiencing hypersensitivity adverse events. Adverse events of hypersensitivity would be reported generally within the SOC “Immune System Disorders.” For these cohorts, within “Immune System Disorders,” the only adverse event considered causally related to investigative product was one subject (IgE negative) who experienced CRS in cohort 5. (One subject in cohort 9 also experienced a causally related CRS.) Notably, no study subject in any cohort experienced any reported hypersensitivity event considered causally related to investigative product.

^f^One ADA negative cohort 8 subject experienced a causally related Infusion-related reaction event, falling within the “Injury, poisoning and procedural complications” SOC.

^g^Investigations comprised of an increase in alanine aminotransferase, aspartate aminotransferase, blood creatine phosphokinase, blood creatinine, gamma-glutamyltransferase, troponin, or a decrease in white blood count.

Furthermore, given the risk of an anti-AMG 256 IgE response based on IL-21 biology and observations in AMG 256 nonclinical studies, subjects were carefully monitored for hypersensitivity reactions. The presence of anti-AMG 256 IgE in two subjects from NCT04362748 confirmed that all the precautions taken in the study design were appropriate. However, there was no apparent impact of the drug-specific IgE on safety (**Table 3**).

## 4 Discussion

Many of the observed effects of AMG 256 in the nonclinical studies in cynomolgus monkeys were consistent with expected pharmacology (15–20). Observations in each AMG 256 study were consistent in their timing (occurring after administration of at least two doses, typically being noted on day 15) and were suggestive of hypersensitivity (including observations of redness, mydriasis, emesis, salivation, decreased activity, coughing, and sneezing). Evidence of complement activation was demonstrated in the exploratory toxicology study, confirming the presence of immune complexes. The administration of biotherapeutics to nonclinical species often leads to the development of immunogenicity and formation of ADAs (21–23); consistent with data reported in these published case studies, observations in monkeys administered AMG 256 included weakness, hunched posture, decreased activity, mydriasis, emesis, hypersalivation, red or pale skin, diarrhea, and loss of consciousness. Clinical pathology changes included reductions in red cell parameters (RBC count, hemoglobin concentration, and hematocrit), thrombocytopenia, prolonged coagulation parameters (PT and aPTT), and acute phase reactions (ie, decreased albumin and increased globulin, increased fibrinogen). Together, these data were indicative of an immunogenicity-related response and are broadly consistent with published case studies.

In the first in human study, a high incidence of anti-AMG 256 antibodies was observed. Based on the amino acid sequence of AMG 256, together with the results of the *in vitro* immunogenicity risk assessment, this was unexpected. The 22D4 clone is a fully human antibody, and the IL-21 mutein domain contains only two point mutations.

While nonclinical studies typically do not predict the risk of immunogenicity in humans (24), in this case, they were informative. Because the fundamental function of IL-21 is similar in cynomolgus monkeys and humans, the IL-21-dependent enhancement of the anti-22D4 antibody response observed in cynomolgus monkey PK/PD and toxicology studies indicated a heightened risk of immunogenicity in humans. While a high incidence of anti-AMG 256 antibodies was observed in NCT04362748, no consequences of this immune response were observed during the study, except for loss of exposure at the two lowest doses.

There are at least 2 potential mechanisms for the unique, dose-dependent immunogenicity observed in NCT04362748. One hypothesis is that when the complementarity-determining regions of the 22D4 domain of AMG 256 are recognized by a B cell, AMG 256 has the potential to both cross-link the BCR receptor and deliver an IL-21 signal, which can lead to plasma cell differentiation (25). Such a mechanism could lead to selective plasma cell expansion of drug-specific B cells. As the dose is increased, cross-linking of the BCR becomes less optimal, and thus the antibody response is mitigated.

An alternative hypothesis is that AMG 256 effectively signals to follicular T helper cells (T_FH_) in the germinal center (GC T_FH_), which highly express PD-1 (26). At low doses of AMG 256, this PD-1-targeted IL-21 signal could lead to enhancement of antibody responses by mediating expansion of GC T_FH_ and/or enhancement of GC T_FH_ activity. At high doses of AMG 256, the IL-21 mutein domain could lead to activation induced cell death of B cells, largely countering the GC T_FH_ -driven mechanism and keeping the antibody response in check (27).

Given the small number of subjects in cohorts 1 and 2, the antibody responses observed may have been due to chance and unrelated to dose. Given the robust nature and unique characteristics of these responses relative to all other dosed subjects, this seems unlikely, but cannot entirely be ruled out without dosing additional subjects at these levels (not feasible for ethical reasons as the dose levels in cohorts 1 and 2 are expected to be sub-efficacious).

While the incidence of ADA often fades as dose increases due to insufficient drug tolerance of the ADA assay, this does not appear to be the case in this study. The ACE method was validated to detect low levels of ADA in the presence of trough levels of AMG 256 throughout the dose escalation cohorts. The nature of the anti-AMG 256 antibody response changed in cohort 3, at which point the levels of AMG 256 in serum are approximately 500-fold below where drug would start to interfere in ADA detection.

AMG 256 represents a rare instance where drug-specific IgE was assessed both nonclinically and in humans using sensitive and drug tolerant assays. The literature on the role of IL-21 and class switching to IgE is mixed with most studies indicating that IL-21 is a negative regulator of IgE class switching (28–31). However, a small set of studies indicate IL-21 can induce IgE secretion (10, 11, 32). Consequently, there are two plausible pathways by which AMG 256 could potentiate a class switch to IgE. One possibility is that in the context of AMG 256 administration, the IL-21 domain directly promotes IgE production. Alternatively, it’s possible that AMG 256 administration elicits an anti-IL-21 antibody response that cross-reacts with and neutralizes IL-21 (a negative regulator of IgE in this case), thereby promoting class switching to IgE. The human IgE data favor the hypothesis that AMG 256-induced IL-21 signaling promotes the switch to IgE, since there was no apparent association between drug-specific IgE and neutralizing antibodies to IL-21.

While drug-specific IgE was detected in both nonclinical and human studies, hypersensitivity was only observed nonclinically. There are several potential reasons for this discrepancy. In cynomolgus monkey studies, only a subset of IgE positive animals developed hypersensitivity. It’s possible hypersensitivity was not observed in NCT04362748 because of the small number of subjects evaluated (2 of 16 subjects were IgE positive). Drug concentrations, levels of anti-AMG 256 IgG (and perhaps other isotypes), and receptor expression levels could all impact the likelihood of type I hypersensitivity, with a reaction only occurring when each of these variables are within a certain range (33). Furthermore, drug-specific IgE was not observed until cycle 6 in NCT04362748, compared to in nonclinical studies where it was observed as early as day 15, suggesting that the underlying mechanism for elicitation of IgE may differ between the cynomolgus monkey and human studies.

Overall, the nonclinical and FIH study data described in this report highlight a novel mechanism of immunogenicity, where a cytokine mutein domain facilitates an ADA response. As protein therapeutics become more complex and incorporate varied functional domains, it will be important to consider all the potential ways each domain can signal to immune cells and contribute to the development of immunogenicity. This MOA-based assessment of immunogenic risk is as important as traditional sequence-based assessments (ie looking for T cell epitopes). This study also yields important insights into how nonclinical studies can, in rare cases, be used to inform on the risk of immunogenicity in humans.

## Data availability statement

Qualified researchers may request data from Amgen clinical studies. Requests to access the datasets should be directed to https://wwwext.amgen.com/science/clinical-trials/clinical-data-transparency-practices/clinical-trial-data-sharing-request/.

## Ethics statement

The studies involving humans were approved by the Institutional Review Board for each study site. The studies were conducted in accordance with the local legislation and institutional requirements. The participants provided their written informed consent to participate in this study. The animal study was approved by Charles River Laboratories Institutional Animal Care and Use Committee. The study was conducted in accordance with the local legislation and institutional requirements.

## Author contributions

MK: Conceptualization, Data curation, Formal analysis, Methodology, Writing – original draft, Writing – review & editing. MM: Conceptualization, Data curation, Methodology, Writing – original draft, Writing – review & editing. CZ: Conceptualization, Data curation, Formal analysis, Investigation, Methodology, Supervision, Writing – original draft, Writing – review & editing. XZ: Conceptualization, Data curation, Formal analysis, Writing – original draft, Writing – review & editing. KC: Conceptualization, Data curation, Formal analysis, Supervision, Writing – original draft, Writing – review & editing. MA: Conceptualization, Data curation, Formal analysis, Writing – original draft, Writing – review & editing. ML: Data curation, Formal analysis, Supervision, Writing – original draft, Writing – review & editing. JW: Data curation, Formal analysis, Methodology, Writing – original draft, Writing – review & editing. SH: Formal analysis, Methodology, Writing – original draft, Writing – review & editing. GS: Data curation, Methodology, Writing – original draft, Writing – review & editing. FA: Conceptualization, Data curation, Formal analysis, Methodology, Writing – original draft, Writing – review & editing. EA: Conceptualization, Data curation, Supervision, Writing – review & editing. EC: Conceptualization, Data curation, Supervision, Writing – review & editing. RG: Conceptualization, Data curation, Supervision, Writing – review & editing. MH: Conceptualization, Data curation, Supervision, Writing – review & editing. SG: Conceptualization, Methodology, Writing – review & editing. DM: Conceptualization, Methodology, Supervision, Writing – original draft, Writing – review & editing.
