# Corrigendum: Translatability of findings from cynomolgus monkey to human suggests a mechanistic role for IL-21 in promoting immunogenicity to an anti-PD-1/IL-21 mutein fusion protein

**DOI:** 10.3389/fimmu.2024.1441999

**Published:** 2024-06-20

**Authors:** Mark A. Kroenke, Marta Starcevic Manning, Christina L. Zuch de Zafra, Xinwen Zhang, Kevin D. Cook, Michael Archer, Martijn P. Lolkema, Jin Wang, Sarah Hoofring, Gurleen Saini, Famke Aeffner, Elizabeth Ahern, Elena Garralda Cabanas, Ramaswamy Govindan, Mun Hui, Shalini Gupta, Daniel T. Mytych

**Affiliations:** ^1^ Clinical Immunology, Amgen, Thousand Oaks, CA, United States; ^2^ Translational Safety & Bioanalytical Sciences, Amgen, Thousand Oaks, CA, United States; ^3^ Translational Safety & Bioanalytical Sciences, Amgen, South San Francisco, CA, United States; ^4^ Clinical Pharmacology, Modeling, and Simulation, Amgen, South San Francisco, CA, United States; ^5^ Pharmacokinetics and Drug Metabolism, Amgen, South San Francisco, CA, United States; ^6^ Global Safety, Amgen, Thousand Oaks, CA, United States; ^7^ Early Development, Amgen, Thousand Oaks, CA, United States; ^8^ Medical Oncology, Monash Health, Clayton, VIC, Australia; ^9^ Research Unit, Hospital Universitario Vall d’Hebron, Barcelona, Spain; ^10^ Division of Hematology and Oncology, Washington University Medical School, St. Louis, MO, United States; ^11^ Chris O’Brien Lifehouse, Camperdown, NSW, Australia

**Keywords:** PD-1, IL-21, immunogenicity, anti-drug antibodies, mutein, IgE

**Error in Figure/Table**

In the published article, there was an error in **Figure 2A** as published. The **Figure 2A** y-axis should read “Serum concentration (ng/mL)”. The corrected **Figure 2** is attached.

**Figure 2:**
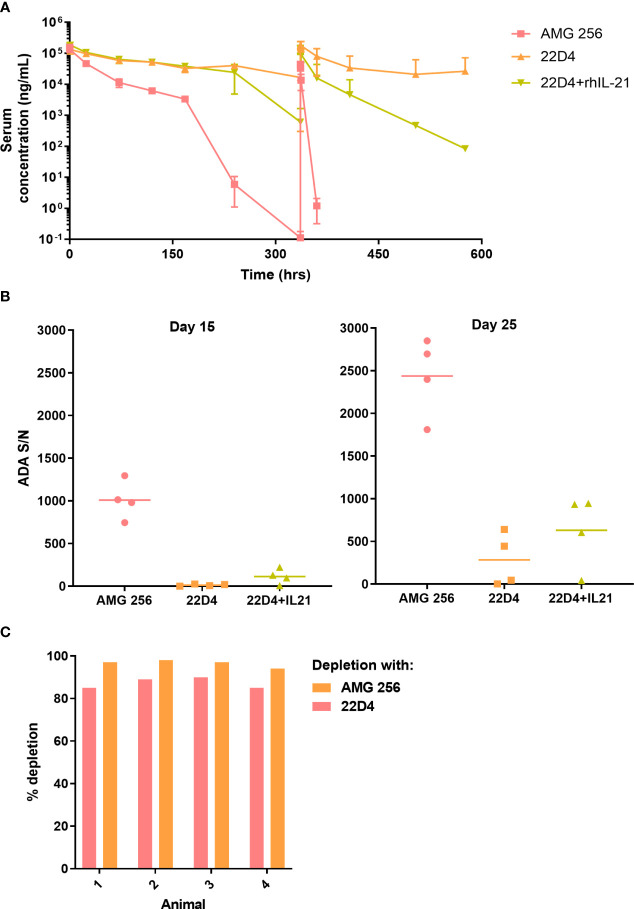
IL-21 mutein domain enhanced the antibody response to 22D4 in cynomolgus monkeys. Cynomolgus monkeys were dosed with 5 mg/kg AMG 256, 5 mg/kg 22D4, or 5 mg/kg 22D4 plus 0.1 mg/kg recombinant human IL-21. **(A)** AMG 256 or 22D4 serum levels were measured over time in each of the 3 treatment groups. **(B)** The ADA response in each dosing group was assessed on day 15 and day 25 by UNISA. **(C)** Domain characterization was performed on AMG 256 dosed animals at the day 25 time point. Serum samples were pre-treated with either AMG 256 or 22D4 and re-tested in the antibody assay. Percent depletion indicates the signal change from the pre-treated sample relative to the untreated sample.

The authors apologize for this error and state that this does not change the scientific conclusions of the article in any way. The original article has been updated.

