# Supplementary material for: Translatability of findings from cynomolgus monkey to human suggests a mechanistic role for IL-21 in promoting immunogenicity to an anti-PD-1/IL-21 mutein fusion protein
Source: Front Immunol. 2024 Jan 26;15:1345473. doi: 10.3389/fimmu.2024.1345473 (PMC10858450; doi:10.3389/fimmu.2024.1345473)
Supplement: Supplementary file 1 [file Table_1.docx]

**Supplemental Table 1. IL-21 Neutralizing Antibody Results for Cohorts 1 and 2**

| Cohort | Dose | Time point | Anti-AMG 256 binding result | IL-21 binding result | IL-21 NAb result |
| --- | --- | --- | --- | --- | --- |
| 1 | 0.6 mg | Cycle 1, Day 1 | Positive | Negative | - |
|  |  | Cycle 1, Day 8 | Positive | Negative | - |
|  |  | Cycle 1, Day 15 | Positive | Negative | - |
|  |  | Cycle 1, Day 22 | Positive | Negative | - |
|  |  | Cycle 2, Day 1 | Positive | Negative | - |
|  |  | Cycle 2, Day 15 | Positive | Positive | Negative |
|  |  | Cycle 3, Day 1 | Positive | Positive | Negative |
|  |  | Cycle 4, Day 1 | Positive | Positive | Positive |
|  |  | Cycle 5, Day 1 | Positive | Positive | Positive |
|  |  | Cycle 6, Day 1 | Positive | Positive | Positive |
|  |  | Cycle 7, Day 1 | Positive | Positive | Positive |
|  |  | Cycle 8, Day 1 | Positive | Positive | Positive |
|  |  | Cycle 9, Day 1 | Positive | Positive | Positive |
|  |  | Cycle 10, Day 1 | Positive | Positive | Positive |
|  |  | Cycle 11, Day 1 | Positive | Positive | Negative |
|  |  | Cycle 12, Day 1 | Positive | Positive | Negative |
|  |  | Cycle 13, Day 1 | Positive | Positive | Negative |
|  |  | Cycle 14, Day 1 | Positive | Positive | Positive |
|  |  | Cycle 15, Day 1 | Positive | Positive | Negative |
|  |  | Cycle 16, Day 1 | Positive | Positive | Positive |
|  |  | Cycle 17, Day 1 | Positive | Positive | Negative |
| 2 | 2 mg | Cycle 1, Day 1 | Negative | - | - |
|  |  | Cycle 1, Day 8 | Negative | - | - |
|  |  | Cycle 1, Day 15 | Positive | Negative | - |
|  |  | Cycle 1, Day 22 | Positive | Negative | - |
|  |  | Cycle 2, Day 1 | Positive | Negative | - |
|  |  | Cycle 2, Day 15 | Positive | Positive | Positive |
|  |  | Cycle 3, Day 1 | Positive | Positive | Positive |
|  |  | Cycle 4, Day 1 | Positive | Positive | Positive |
|  |  | Cycle 5, Day 1 | Positive | Positive | Positive |
|  |  | Cycle 6, Day 1 | Positive | Positive | Positive |
|  |  | End of IP | Positive | Positive | Positive |
