# Supplementary material for: Translatability of findings from cynomolgus monkey to human suggests a mechanistic role for IL-21 in promoting immunogenicity to an anti-PD-1/IL-21 mutein fusion protein
Source: Front Immunol. 2024 Jan 26;15:1345473. doi: 10.3389/fimmu.2024.1345473 (PMC10858450; doi:10.3389/fimmu.2024.1345473)
Supplement: Supplementary file 2 [file Table_2.docx]

**Supplemental Table 2. Samples tested in anti-AMG 256 IgE assay**

| Cohort | Dose | Subject ID | Time point | Anti-AMG 256 IgE result |
| --- | --- | --- | --- | --- |
| 1 | 0.6 mg QW | 14466004001 | Cycle 1, Day 1 | Negative |
|  |  |  | Cycle 1, Day 8 | Negative |
|  |  |  | Cycle 1, Day 15 | Negative |
|  |  |  | Cycle 2, Day 1 | Negative |
|  |  |  | Cycle 6, Day 1 | Positive |
|  |  |  | Cycle 12, Day 1 | Positive |
| 2 | 2 mg QW | 1441005001 | Cycle 1, Day 1 | Negative |
|  |  |  | Cycle 1, Day 8 | Negative |
|  |  |  | Cycle 1, Day 15 | Negative |
|  |  |  | Cycle 2, Day 1 | Negative |
|  |  |  | Cycle 6, Day 1 | Negative |
| 3 | 6 mg QW | 14411004002 | Cycle 1, Day 1 | Negative |
|  |  |  | Cycle 1, Day 8 | Negative |
|  |  |  | Cycle 1, Day 15 | Negative |
|  |  |  | Cycle 2, Day 1 | Negative |
|  |  |  | Cycle 6, Day 1 | Negative |
|  |  |  | Cycle 12, Day 1 | Negative |
| 4 | 20 mg QW | 14411005004 | Cycle 1, Day 1 | Negative |
|  |  |  | Cycle 1, Day 8 | Negative |
|  |  |  | Cycle 2, Day 1 | Negative |
|  |  |  | EOIP (Day X) | Negative |
|  |  | 14411004004 | Cycle 1, Day 1 | Negative |
|  |  |  | Cycle 1, Day 8 | Negative |
|  |  |  | Cycle 1, Day 15 | Negative |
|  |  |  | Cycle 2, Day 1 | Negative |
|  |  |  | Cycle 6, Day 1 | Negative |
|  |  |  | Cycle 12, Day 1 | Negative |
| 5 | 60 mg QW | 14411005006 | Cycle 1, Day 1 | Negative |
|  |  |  | Cycle 1, Day 8 | Negative |
|  |  |  | Cycle 1, Day 15 | Negative |
|  |  |  | Cycle 2, Day 1 | Negative |
|  |  |  | Cycle 6, Day 1 | Positive |
|  |  |  | Cycle 12, Day 1 | Positive |
|  |  | 14411004006 | Cycle 1, Day 1 | Negative |
|  |  |  | Cycle 1, Day 8 | Negative |
|  |  |  | Cycle 1, Day 15 | Negative |
|  |  |  | Cycle 2, Day 1 | Negative |
|  |  |  | EOPI (Day X) | Negative |
|  |  | 14411003001 | Cycle 1, Day 1 | Negative |
|  |  |  | Cycle 1, Day 8 | Negative |
|  |  |  | Cycle 1, Day 15 | Negative |
|  |  |  | Cycle 2, Day 1 | Negative |
|  |  |  | EOPI (Day X) | Negative |

**Supplemental Table 2 (continued)**

| Cohort | Dose | Subject ID | Time point | Anti-AMG 256 IgE result |
| --- | --- | --- | --- | --- |
| 7 | 500 mg QW | 14411003004 | Cycle 1, Day 1 | Negative |
|  |  |  | Cycle 1, Day 8 | Negative |
|  |  | 14458002003 | Cycle 1, Day 1 | Negative |
|  |  |  | Cycle 1, Day 8 | Negative |
|  |  |  | Cycle 1, Day 15 | Negative |
|  |  |  | Cycle 2, Day 1 | Negative |
|  |  | 14458002002 | Cycle 1, Day 1 | Negative |
|  |  |  | Cycle 1, Day 8 | Negative |
|  |  |  | Cycle 1, Day 15 | Negative |
|  |  |  | Cycle 2, Day 1 | Negative |
|  |  | 14458001003 | Cycle 1, Day 1 | Negative |
|  |  |  | Cycle 1, Day 8 | Negative |
|  |  |  | Cycle 1, Day 15 | Negative |
|  |  |  | Cycle 2, Day 1 | Negative |
| 1a | 1000 mg Q2W | 14411004007 | Cycle 1, Day 1 | Negative |
|  |  |  | Cycle 1, Day 15 | Negative |
|  |  |  | Cycle 2, Day 1 | Negative |
|  |  | 14466004002 | Cycle 1, Day 1 | Negative |
|  |  |  | Cycle 1, Day 15 | Negative |
|  |  |  | Cycle 2, Day 1 | Negative |
|  |  | 14458001006 | Cycle 1, Day 1 | Negative |
|  |  |  | Cycle 1, Day 15 | Negative |
|  |  |  | Cycle 2, Day 1 | Negative |
|  |  | 14458001005 | Cycle 1, Day 1 | Negative |
|  |  |  | Cycle 1, Day 15 | Negative |
|  |  |  | Cycle 2, Day 1 | Negative |
